# From rags to enriched: metagenomic insights into ammonia‐oxidizing archaea following ammonia enrichment of a denuded oligotrophic soil ecosystem

**DOI:** 10.1111/1462-2920.15994

**Published:** 2022-04-15

**Authors:** Paton Vuong, Benjamin Moreira‐Grez, Michael J. Wise, Andrew S. Whiteley, Deepak Kumaresan, Parwinder Kaur

**Affiliations:** ^1^ UWA School of Agriculture & Environment University of Western Australia Perth Australia; ^2^ School of Physics, Mathematics and Computing University of Western Australia Perth Australia; ^3^ The Marshall Centre of Infectious Diseases School of Biological Sciences, The University of Western Australia Perth Australia; ^4^ Centre for Environment & Life Sciences Commonwealth Scientific and Industrial Research Organisation (CSIRO) Floreat Australia; ^5^ School of Biological Sciences Queen's University of Belfast Belfast UK

## Abstract

Stored topsoil acts as a microbial inoculant for ecological restoration of land after disturbance, but the altered circumstances frequently create unfavourable conditions for microbial survival. Nitrogen cycling is a critical indicator for ecological success and this study aimed to investigate the cornerstone taxa driving the process. Previous *in silico* studies investigating stored topsoil discovered persistent archaeal taxa with the potential for re‐establishing ecological activity. Ammonia oxidization is the limiting step in nitrification and as such, ammonia‐oxidizing archaea (AOA) can be considered one of the gatekeepers for the re‐establishment of the nitrogen cycle in disturbed soils. Semi‐arid soil samples were enriched with ammonium sulfate to promote the selective enrichment of ammonia oxidizers for targeted genomic recovery, and to investigate the microbial response of the microcosm to nitrogen input. Ammonia addition produced an increase in AOA population, particularly within the genus *Candidatus Nitrosotalea*, from which metagenome‐assembled genomes (MAGs) were successfully recovered. The *Ca*. *Nitrosotalea* archaeon candidates' ability to survive in extreme conditions and rapidly respond to ammonia input makes it a potential bioprospecting target for application in ecological restoration of semi‐arid soils and the recovered MAGs provide a metabolic blueprint for developing potential strategies towards isolation of these acclimated candidates.

## Introduction

Mining operations often involve the clearing of vegetation, and removal and subsequent storage of topsoil, which serves as a critical reserve of soil microbial communities for post‐mining rehabilitation and restoration of ecosystem processes (Williams *et al*., 2019). Long‐term storage has been shown to change community structure and diversity, which may have negative implications for nutrient cycling and other vital ecological processes (Ezeokoli *et al*., 2019). As soil microbial populations are essential for plant community development, successful restoration of degraded terrestrial ecosystems requires the input of effective soil inoculum (Wubs *et al*., 2016). In determining the microbial diversity and functional capacity of stored topsoil and substrate blends (topsoil and mine site waste substrates) in mine site restoration scenarios in Western Australian (WA) conditions, we showed phylogenetic divergence but ‘potential’ functional redundancy for microbe‐related functions and reported enrichment of archaeal taxa across different topsoil blends, indicating a persistent group of taxa (Kumaresan *et al*., 2017). Long‐term storage of topsoil can contribute to loss of the seed bank, leading to the decline of the re‐emergence of native and endemic flora (Golos and Dixon, 2014). In a follow‐up study, we also showed that biocrusts, the aggregated layer containing crucial microbial community and the majority of biological activity within arid soils, can play an important role in nitrogen cycling within arid landscapes in WA soils (Moreira‐Grez *et al*., 2019b). Soil biodiversity is key for ecosystem multifunctionality (Wagg *et al*., 2014) and ensuring microbial functional diversity in mine site restoration scenario is critical to enable critical nutrient cycling processes.

Nitrogen is considered a major limiting nutrient for plant growth within terrestrial ecosystems, and microbial processes facilitate availability of nitrogen compounds for plant growth, i.e. nitrogen fixation, ammonia oxidation (Moreau *et al*., 2019). In the nitrification step of nitrogen cycling, ammonia oxidation is the initial and limiting step and is performed by ammonia‐oxidizing archaea (AOA), ammonia‐oxidizing bacteria (AOB) and complete ammonia oxidation (comammox) bacteria (Kuypers *et al*., 2018; Lehtovirta‐Morley, 2018). As such, these ammonia‐oxidizing taxa are arguably the gatekeepers of nitrogen cycling and understanding their ecological presence is key to determining the sustainability of an ecosystem (Amoo and Babalola, 2017). AOA, in comparison to AOB, dominate soil microbiomes with low ammonium supply. The metabolic and ecological differences between archaeal and bacterial ammonia oxidizers that modulate this niche partitioning have been previously described in dryland soils (Trivedi *et al*., 2019). For instance, within the weathered lateritic agricultural WA soils, a reverse trend has been observed, where AOB are present in higher numbers compared to AOA. The authors speculate that the low copper levels (a key co‐factor ammonia oxidation in AOA) in WA agricultural soils could drive the functions (Jenkins *et al*., 2016). WA soils could be host to unique microbial communities that are adapted to the lateritic soils, which are often depleted of other nutrients, such as phosphorus, potassium and critically, nitrogen, due to extensive weathering and the binding of nutrients to lateritic compounds that make them unavailable for uptake (O'Brien *et al*., 2019).

In our previous work, we highlighted the higher relative abundance of novel 16S rRNA gene sequences related to archaeal taxa involved in nitrogen cycling. Unlike agricultural soils in WA (Jenkins *et al*., 2016), the nitrifiers in natural ecosystems, particularly rich in iron‐ore deposits are largely unknown. Shotgun metagenome sequencing has the potential to pinpoint cornerstone taxa that may play critical roles throughout the ecological restoration due to their capacity to link both taxonomic and functional information (Hart *et al*., 2020). Here, we aim to recover genomic data of ammonia‐oxidizing taxa from ammonium‐enriched stored topsoil from the mining sites in semi‐arid WA soils, with particular focus on the persistent archaeal presence observed in our previous work (Kumaresan *et al*., 2017; Moreira‐Grez *et al*., 2019a). Enrichment of the microcosm using inorganic ammonium coupled with metagenomic sequencing led to improved genomic recovery of AOA that provided *in silico* blueprints of the metabolic capacity of recovered candidates that can aid in developing isolation strategies for potential bioprospecting ventures. The enrichment was also used to compare the response of AOA and AOB groups to ammonium input in oligotrophic conditions. The metagenomic approach utilized in this study provided insight into the ecological drivers of ammonia‐oxidizing taxa in the semi‐arid soils from natural ecosystems in WA, with a focus towards their nitrogen cycling potential in the restoration of disturbed soils.

## Results

### Temporal taxonomic profiles of the soil metagenomes

**Fig. 1:**
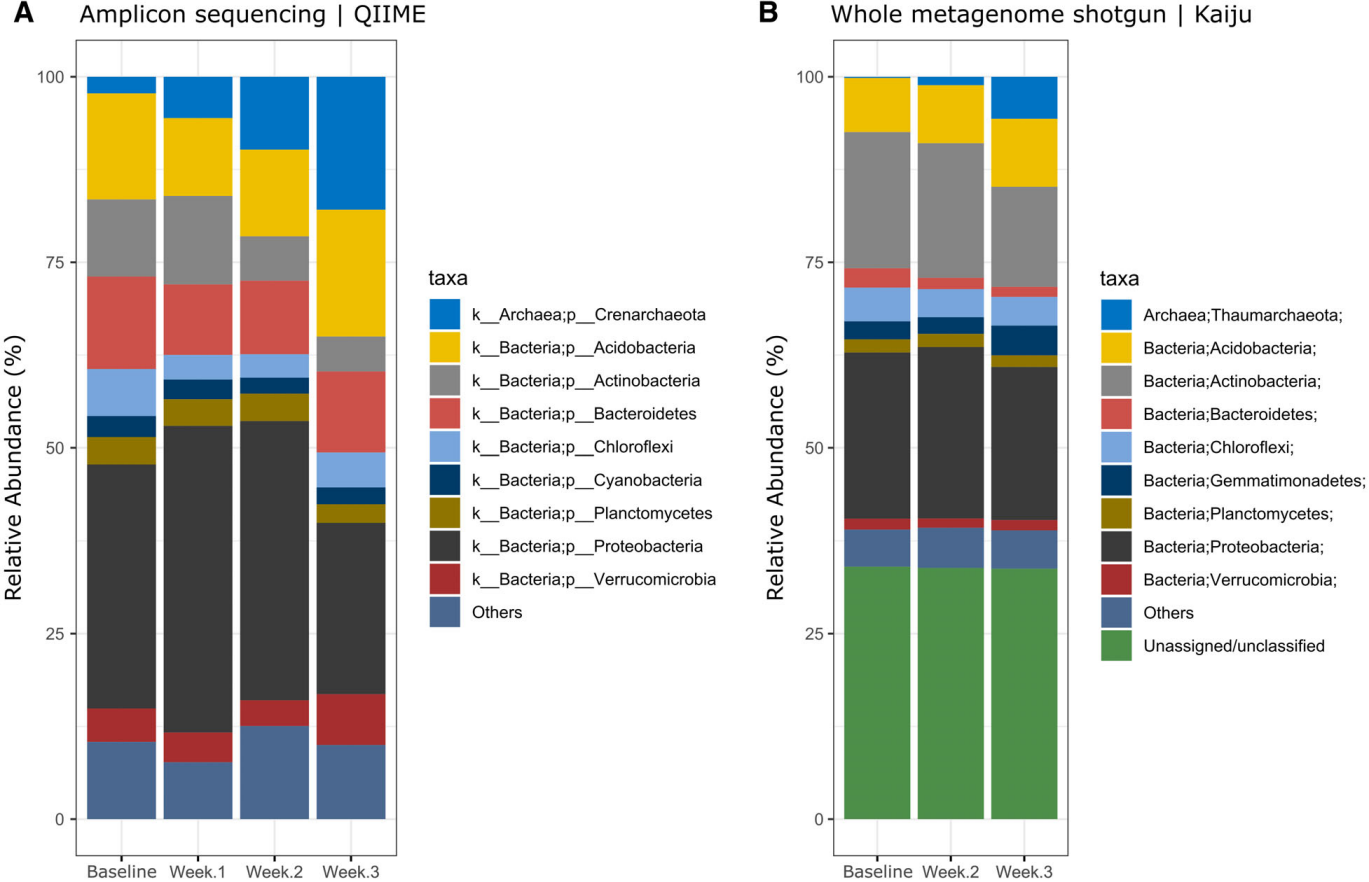
Taxonomic distribution at the phylum level of the soil metagenome from amplicon and whole metagenome shotgun sequencing data. Comparisons between baseline soil and time points post‐enrichment with ammonium sulfate. Amplicon sequencing data were used solely to determine relevant time points for the subsequent whole metagenome shotgun sequencing.

**Fig. 2:**
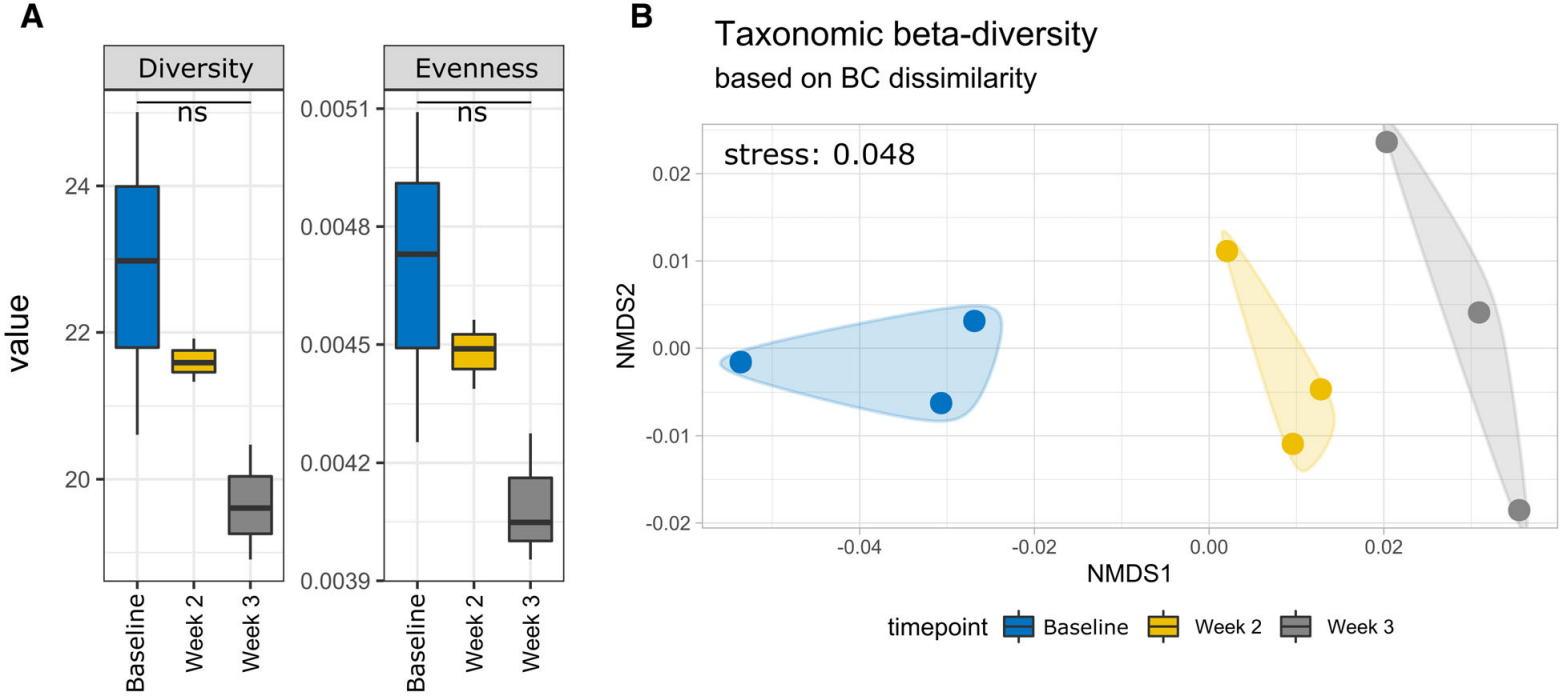
Measurements of microbial community structure at the genus level from Kaiju outputs using whole metagenome shotgun data. Baseline and treatment populations were investigated for temporal changes in (A) Alpha‐diversity though Shannon diversity index with differences between samples determined to be not significant via one‐way ANOVA and (B) Beta‐diversity via NMDS of microbial community dissimilarity.

Semi‐arid soils subjected to enrichment with (NH_4_)_2_SO_4_ showed similar proportions in the overall bacterial populations, with only slight variation in relative abundance observed across time points between both the amplicon (Fig. 1A) and whole metagenome shotgun (WMS) data (Fig. 1B). The most substantial increase in relative abundance after 3 weeks of N enrichment were sequences related to archaeal phyla Thaumarchaeota, Aigarchaeota, Crenarchaeota and Korarchaeota superphylum (Guy and Ettema, 2011) which contains the canonical AOA. This is most notable in the Archaea; Thaumarchaeota phylum from the WMS data, with an increase in relative abundance from 0.155% (baseline) to 5.629% (3 weeks), a roughly 34‐fold increase in metagenomic reads classified as Thaumarchaeota. The disparity between the phylum naming for the amplicon sequences and WMS data is likely due to the differences in the respective databases utilized by each method. The publicly available versions of the Greengenes 16S rRNA gene database use older taxonomic terms, likely from when Thaumarchaeota was still classified as a ‘mesophilic Crenarchaeota’ (Pester *et al*., 2011). As the WMS classification is based on the latest NBCI non‐redundant protein database, it provides a more up to date classification consistent with current literature. Another point of note is the replacement of Cyanobacteria from amplicon sequences to Gemmatimonadetes in the WMS data, most likely due to misidentification of chloroplast sequences among the 16S amplicons (Hanshew *et al*., 2013). The diversity and evenness of the soil samples were shown to decrease over time post‐enrichment, likely due to the substantial change in relative abundance observed within the archaeal phyla; however, this change was determined to be statistically not significant according to one‐way ANOVA (Fig. 2A). The clustering of the soil sample data in the non‐metric multidimensional scaling (NMDS) analysis showed a clear successional trend observed in the microbial communities as time progressed post (NH_4_)_2_SO_4_ enrichment (Fig. 2B).

### Effects of enrichment on ammonia‐oxidizing prokaryote populations

**Fig. 3:**
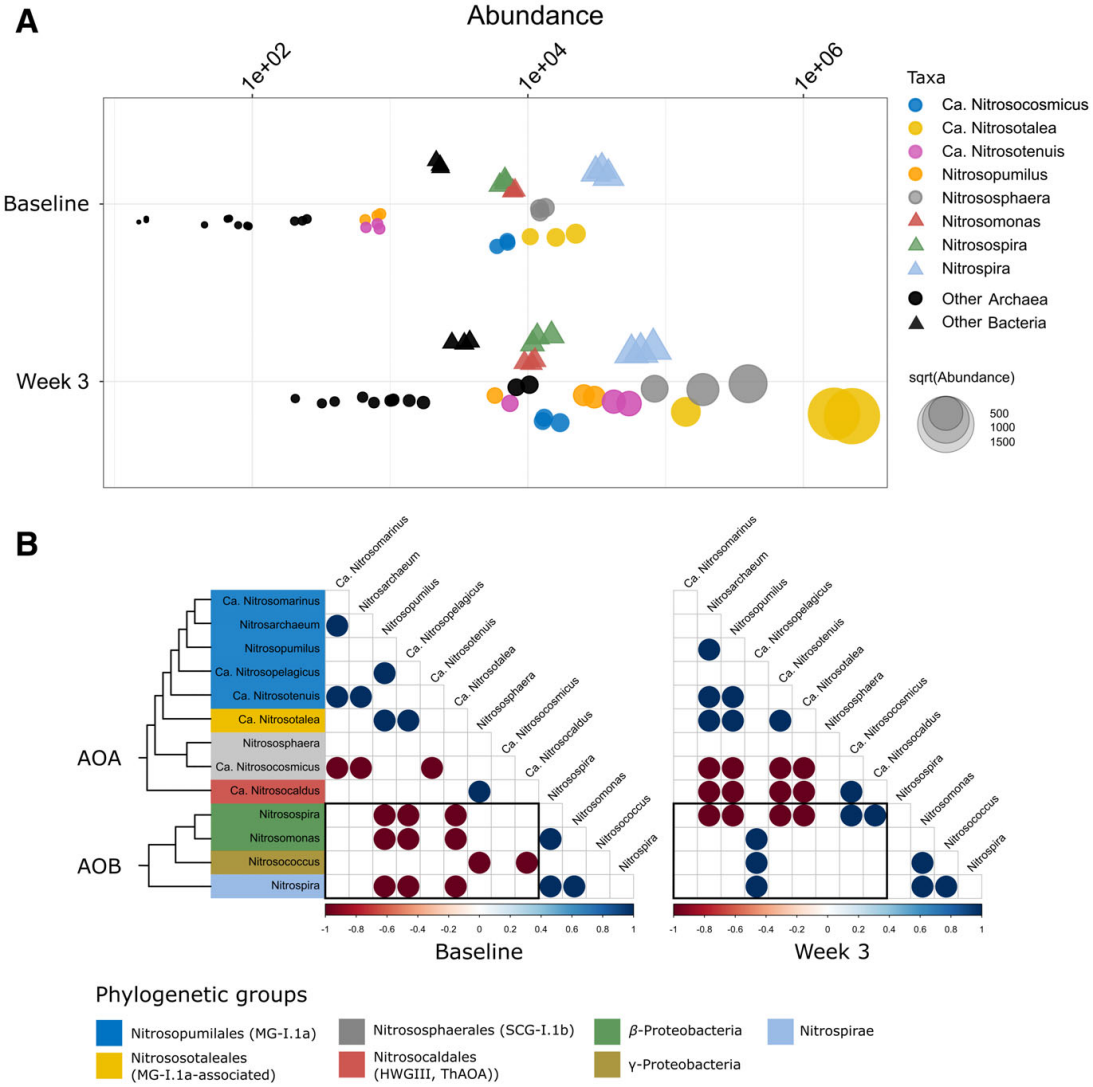
Taxonomic profiling of a subset of ammonia‐oxidizing taxa at the genus level from soil metagenome. A. Relative counts of archaea (circles) and bacteria (triangles) from baseline and 3‐week post‐ammonia sulfate enrichment. B. Correlation plot of AOA versus AOB (including *Nitrospira*). Area within the rectangle seen in the correlation plot highlights all archaea–bacteria associations.

Sequences affiliated to AOA showed greater increase in relative abundance compared to AOB at the genus level across successive time points post‐enrichment with (NH_4_)_2_SO_4_ (Fig. 3A). In the baseline soils, AOA abundances were either at similar levels or lower than those of AOB with respect to relative proportion within the overall population. Over the course of the experimental period, the relative abundances of all detected AOA increased, whereas the AOB population remained similar or showed only slight increases on a subset of taxa. At the end of the 3‐week period, AOA from the genus *Candidatus Nitrosotalea* and *Nitrososphaera* showed higher relative abundances across all samples, with *Ca*. *Nitrosotalea* showing at least a 10‐fold increase in relative abundance within two replicate samples compared to the highest reported AOB genus. *Nitrospira* showed a marginal increase at week 3; however, as no MAGs were recovered from this genus, we were unable to determine if the reported populations were composed of comammox or nitrite oxidizers. Correlations of ammonia‐oxidizing taxa showed strong associations between the pairs of AOA and AOB (Fig. 3B). In the baseline soils, multiple bacterial and archaeal taxa had a strong negative correlation, indicating competitive interactions between both groups. After 3 weeks, aside from *Nitrosospira*, these associations were no longer observed between archaeal and bacterial groups. Coupled with the substantial increase seen in AOA but not AOB numbers in the relative abundance data suggests that within this subset of ammonia‐oxidizing prokaryotes, archaea may have out‐competed most bacteria groups after 3 weeks post‐enrichment.

### Differential abundances of the metabolic profile of the metagenome post‐enrichment

**Fig. 4:**
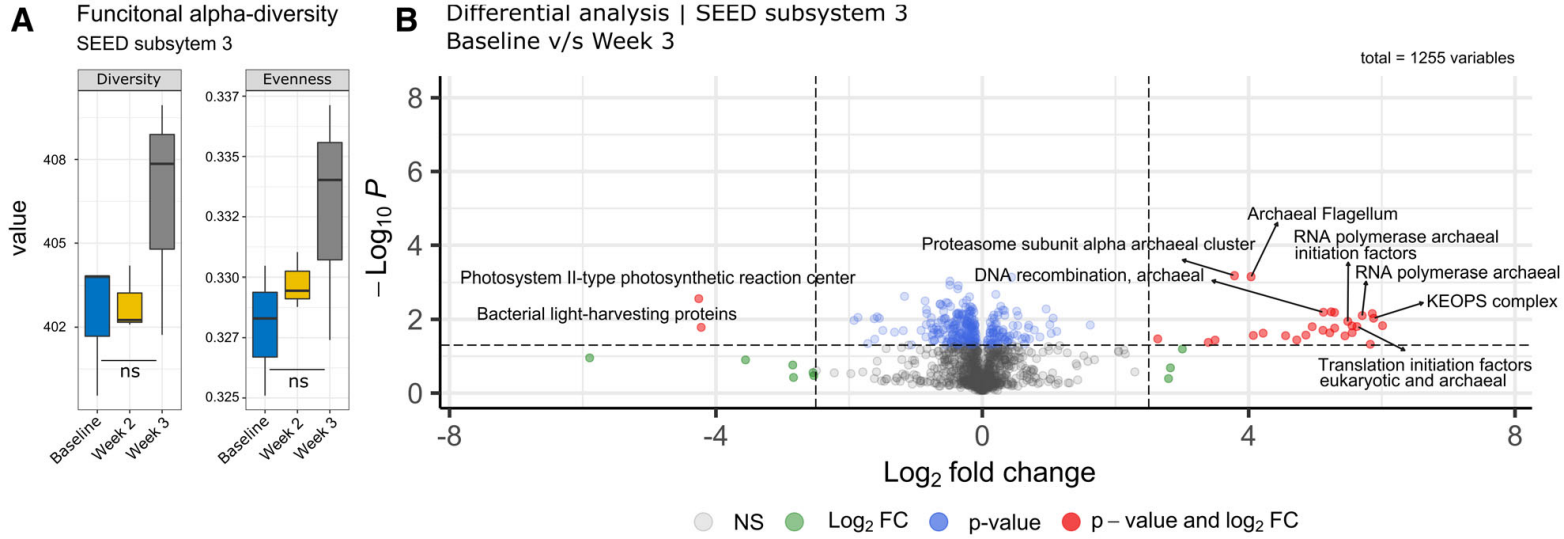
Changes in metabolic gene distributions at SEED subsystem level 3 observed in the baseline and post ammonium sulfate enriched soils. A. Box plot of Shannon diversity and evenness index of metabolic genes showing overall changes from all time points. One‐way ANOVA determined differences between samples were not significant. B. Volcano Plot produced from ALDEx2 analysis of metabolic genes from baseline samples versus 3‐weeks post‐enrichment. Data points in red indicate that the metabolic gene in question was either significantly enriched or suppressed in comparison to others according to Welch's *t* and Wilcoxon rank test (*α* < 0.05).

At the level 3 subsystem category, the overall diversity and evenness of metabolic genes displayed a marginal increase across all soil samples as time progressed post‐enrichment; however, the one‐way ANOVA testing determined that the changes observed were determined to be not significant (Fig. 4A). In comparison, certain individual metabolic genes within the level 3 subcategory were significantly enriched or suppressed in the 3‐week post‐enrichment treatment sample compared to the baseline soil (Fig. 4B, Supplementary Table 1). The genes of interest that were significantly enriched were those associated with archaeal biosynthesis and transcription. This pattern of diversity is also reflected in the taxonomic abundance data, in which the overall population showed little to marginal change, but substantial increases were seen in sequences associated with archaeal taxa.

### Archaeal genomes recovered from soil metagenome

A total of five archaeal MAGs were recovered from across all soil metagenomes that passed the completeness and contamination protocols. As GTDB‐tk was used to classify the MAGs, the GTDB naming convention will be used to categorize the taxonomic labels for all resultant data involving the MAGs recovered in the study. Four MAGs were classified from canonical AOA genera, three from genus *Nitrosotalea* (CheckM reported completeness – nc_117: 64.56%, t2_109: 58.90% and t3_48: 92.23%) and one from genus *Nitrososphaera* (completeness – t2_80: 78.80%). The remaining MAG was an archaeon from order Woesearchaeales. Additional information on all recovered MAGs such as quality and assembly statistics, assigned classification, classification method, predicted tRNAs and rRNA can be found in Supplementary Table 2.

### Effects of enrichment on diversity capture and sequence utilization in metagenomic assemblies

**Fig. 5:**
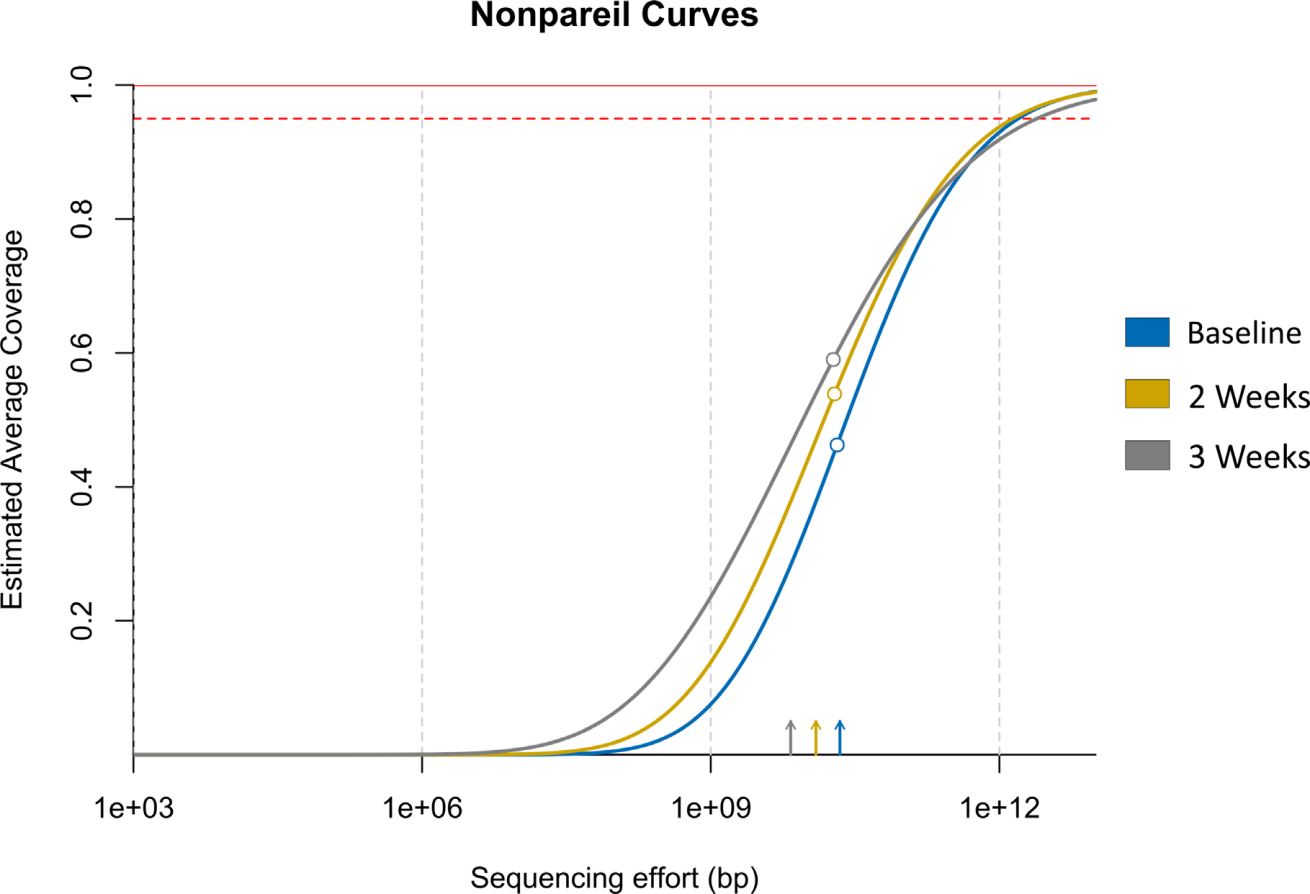
Estimation of the coverage of diversity between the baseline and post‐enrichment soil metagenomes using Nonpareil. The empty circles on the curves show the actual sequencing volume of the respective datasets. The dashed and solid red horizontal lines show the 95% and 100% estimated coverage threshold respectively. The arrows are a visual indicator of the Nonpareil diversity index of each dataset, with arrows further right indicating more diverse communities.

The redundancy estimation by Nonpareil showed increased rates of coverage for the diversity observed within the post‐enrichment soil metagenome compared to that of the baseline soils, given similar sequencing efforts (Fig. 5). The improved coverage likely reflects the decreased diversity observed in the post‐enrichment metagenomes (Fig. 2A), which is also supported by the values reported by the Nonpareil diversity index (Baseline: 23.81, 2 weeks: 23.24, 3 weeks: 22.64). Read mapping of the baseline, 2‐week and 3‐week metagenomic reads to their respective assembled sequences reported an overall alignment rate of 46.69%, 53.51% and 57.71% respectively, which closely mirrors the values presented by the estimated average coverage seen on the Nonpareil curves to their respective datasets. Improvements in overall alignment rates from the post‐enrichment metagenomes were also seen downstream in the recovered AOA candidate MAGs, with a clear pattern observed in the *Nitrosotalea* candidates, nc_117 (baseline): 0.02%, t2_109 (2 weeks): 0.07% and t3_48 (3 weeks): 0.17%, along with the sole *Nitrososphaera* candidate MAG t2_80 (2 weeks): 0.29%. The increased alignment rates in the post‐enrichment metagenomes and MAGs demonstrate that more reads are being utilized in assembled sequences, likely due to the reduction in diversity resulting in better coverage. Increased read maps in the *Nitrosotalea* candidate MAGs from successional metagenomes also suggest that the more abundant *Nitrosotalea* subpopulations (Fig. 3A) observed in the post‐enrichment microbiomes have improved the genomic capture rate of this taxa.

### Phylogenomic comparison and distributions of nitrogen metabolism pathways of interest

**Fig. 6:**
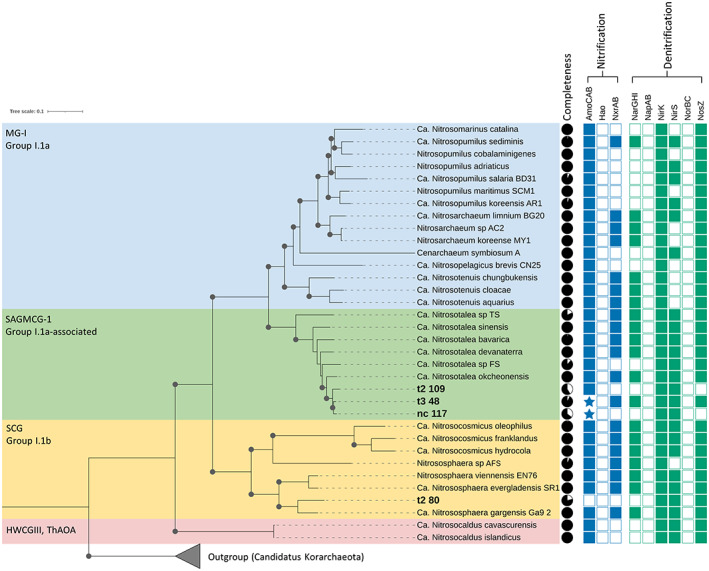
Maximum likelihood tree and nitrogen pathways of archaeal genomes. AOA genomes from RefSeq were phylogenetically compared to the potential AOA MAGs recovered from this study (indicated by bold labels). Completeness of MAGs was reported by CheckM. Nitrogen pathways displayed were predicted by MinPath using the KEGG metabolic pathway models and are represented by shaded boxes indicating the predicted presence of a node within the respective nitrogen metabolic pathway. Shaded stars are nodes that were not present in the MinPath prediction but were detected via HMM search using the Pfam A database. Bootstrap values of ≥95% are shown by solid dots. Coloured backgrounds indicate AOA groups: Marine Group I (MG‐I); South African Gold Mine Crenarchaeotic Group 1 (SAGMCG‐1); Soil Crenarchaeotic Group (SCG); Hot Water Crenarchaeotic Group III (HWCGIII) and Thermophilic AOA (ThAOA).

The four archaeal MAGs classified as AOA candidates appeared to be closely related to the known ammonia‐oxidizers *Nitrososphaera* and *Nitrosotalea*, which is congruent with the genus classification assigned by GTDB‐tk (Fig. 6). All the MAGs identified as genus *Nitrosotalea* (nc_117, t2_109 and t3_48) were found to contain *amoA* and the t3_48 MAG which had the highest completion, showed a nitrogen metabolic pathway pattern consistent with several *Nitrosotalea* and other AOA genomes. Although the genus *Nitrososphaera* MAG (t2_80) was phylogenetically assigned within the *Nitrososphaera* clade, it is missing the AmoCAB node or results from Pfam/TIGRFAM searches required to definitively confirm it as an AOA. Given the phylogenetic placement, this is most likely due to missing data resulting from the MAG being incomplete, rather than an actual absence of ammonia monooxygenase.

## Discussion

Metagenomics provides a primary approach into the observations of the *in situ* metabolic potential of environmental microorganisms and their response to ecological changes. Furthermore, the recovery of MAGs allows us to understand the metabolic capacity of a microbiome within the context of the individual microorganism present. The prediction of metabolic potential, however, does not automatically guarantee function. A pertinent example is the denitrification process where varying ecological factors determine whether transcription occurs or even if functional enzymes are produced, despite the presence of denitrification genes within the genome (Lycus *et al*., 2017). Another issue is that due to the limitations of short‐read sequencing, the MAGs often remain drafts, with the missing data obscuring valuable ecological and functional information. Nonetheless, extensive *in silico* approaches such as this study provide the groundwork to explicitly select candidate taxa for potential bioprospecting ventures and the ability to further explore other biosynthetic pathways present, within the context of the ecological question at hand.

From the denuded environment present in the stored topsoil, archaea appeared to have displayed the greatest relative response to the (NH_4_)_2_SO_4_ enrichment. This is reflected in the significant increases seen within Thaumarchaeota at the phylum level, AOA at the genus level, as well as in archaeal‐associated metabolic gene groups among the metagenomic reads. Studies into archaeal involvement in plant–microbe interactions have shown that not only do archaea participate in nutrient cycling but also promote growth, improve disease resistance and mitigate stress factors in plants (Jung *et al*., 2020). This suggests that stored topsoil treated with (NH_4_)_2_SO_4_ to stimulate the growth of AOA populations can be used to improve soils for post‐land‐use rehabilitation.

In this pilot study, we investigated the potential of using inorganic nitrogen addition within the context of soil restoration and how it can modulate nitrifier activity/diversity. However, the experiments on the basal soils did not account for the growth of AOAs without enrichment. Further AOA propagation studies within stored topsoil should consider baseline community succession, i.e. without nutrient input, to account for other potential ecological interactions within the microcosm. Future field‐scale trials will certainly account for other contributing factors such as nitrogen fixation, plant–microbe interactions, among other variables, that can modulate nitrogen availability. Here, our focus was to identify keystone taxa and recover the genomes of indigenous archaeal nitrifiers.

Variations in the observed AOA and AOB population numbers between baseline and enriched soils provided valuable insight into the potential effects of N addition within natural ecosystems in WA. Short‐term addition of inorganic ammonia in the form of (NH_4_)_2_SO_4_ appeared to promote the rapid growth of AOA in the semi‐arid topsoil. The large increase in AOA population numbers compared to AOB post‐(NH_4_)_2_SO_4_ enrichment may be due to the inhibitory effect of high ammonia concentrations, which has been observed to affect the growth of AOB but not AOA (Liang *et al*., 2020). This niche differentiation of AOA and AOB could be utilized when developing fertilization strategies for the promotion of AOA growth for nitrification within the semi‐arid soils present in WA.

Further studies exploring ammonia‐oxidizing taxa are crucial for understanding the ecological processes that drive nitrifying microorganisms within the WA natural soil ecosystems. Ecological studies of ammonia‐oxidizers have been carried out in managed soils in WA, particularly within agricultural soils (O'Sullivan *et al*., 2013; Banning *et al*., 2015; Fisk *et al*., 2015; Jenkins *et al*., 2016), but very little work has been done in native terrestrial environments. A thorough understanding of the edaphic properties of soil in natural ecosystems and how they affect ammonia‐oxidizing taxa are essential for the restoration of disturbed soils to their state prior to land use. This process is vital for screening ideal candidate taxa acclimated to the ecological conditions present within the unique soil ecosystems present within the natural WA landscape.

Within the dominant post‐enrichment AOA population, the genus *Ca*. *Nitrosotalea* displayed the greatest relative abundance in archaeal reads and provided the highest number of recovered archaeal MAGs. The *Ca*. *Nitrosotalea* archaeon thrive in acidic environments (pH 4.5–5.5) through high‐affinity substrate acquisition and pH homeostasis systems which confer the ability to oxidize ammonia at low pH, conditions that have been found to inhibit AOB activities (Lehtovirta‐Morley *et al*., 2016a). High rates of horizontal gene transfer discovered in other *Ca*. *Nitrosotalea* genomes likely contributed to the lineage's ability to be successful in extreme environments, by facilitating the procurement of physiological systems allowing for survival in harsh environments (Herbold *et al*., 2017). The properties associated with *Ca*. *Nitrosotalea* likely contributed to the relative success in propagation observed in the candidate taxa found within this study, as the stored topsoil was derived from acidic soils (Kumaresan *et al*., 2017).

AOA that can persist in extreme conditions are desirable bioprospecting targets for isolation as they can provide effective soil remediation in a broader range of ecological conditions. *Candidatus Nitrosocosmicus franklandus*, an AOA species isolated from arable soil, has been observed to thrive in high ammonia concentrations potentially providing contributions towards the nitrification of fertilized soils due to its physiological diversity (Lehtovirta‐Morley *et al*., 2016b). The *Ca*. *Nitrosotalea* candidates sequenced in this study comparatively have also been shown to respond well to high ammonium input but are able to survive in denuded oligotrophic conditions commonly seen in stored topsoil. Combined with the known *Ca*. *Nitrosotalea* archaeon ability to survive in acidic environments makes the *Ca*. *Nitrosotalea* candidates discovered in this study prime target for isolation and utilization as indigenous microorganism for the restoration of semi‐arid soil ecosystems present in WA. Novel isolation or *in situ* enrichment techniques that facilitate the inoculation of *Ca*. *Nitrosotalea* can potentially speed up the re‐establishment of the N‐cycle upon disturbed soils.

In tandem, AOA produce less N_2_O emissions compared to AOB under both high and low ammonium supply, making AOA ideal candidates for the reduction in N_2_O production (Hink *et al*., 2018). Within terrestrial environments, excess use of nitrogen‐based fertilizer has led to an increase in the production of nitrous oxide (N_2_O), a major greenhouse gas, from nitrifying and denitrifying microbes (Thompson *et al*., 2019). There has been increased interest in investigating the physiology and niche differentiation in AOA, AOB and comammox bacteria to develop strategies that optimize microbial processes within the nitrogen cycle, whilst mitigating the effects of greenhouse gas production (Prosser *et al*., 2020). Microbial bioprospecting of natural soil ecosystems with low nitrogen content, such as oligotrophic soil ecosystems could produce candidate AOA that can potentially operate under conditions with minimal nitrogen input and may further reduce N_2_O production.

## Conclusion

Stored topsoil is vital for restoring post‐land‐use ecosystems and understanding the microbial communities that persist within the less‐than‐ideal conditions during storage is crucial for successful rehabilitation. Metagenomic approaches coupled with the enrichment of the topsoil biome have provided insight on taxa involved with critical nitrogen cycling capacity that can persist in the denuded oligotrophic conditions, as well as those that can respond rapidly to nitrogen input. Through MAGs, we were able to further explore metabolic capacity within the context of taxa allowing us to explicitly shortlist candidate taxa with diverse nitrogen metabolism. These metagenomic insights can be applied to develop strategies in bioprospecting for acclimated taxa that can perform essential ecological functions needed to revitalize degraded ecosystems. In addition, further insight into the nitrogen metabolism in nitrifying and denitrifying taxa in different ecological niches may aid in the understanding of microbial N_2_O production in a bid to reduce climate footprints by finding suitable candidates for use in anthropogenic activities.

## Experimental procedures

### Sample collection

Soil samples were collected from topsoil storage from an active mine site 200 km east of Geraldton, WA (−29.164658°, 116.786696°). Soil collection protocols are detailed under the ‘Materials and Methods’ section in the study by Kumaresan *et al*. (2017), with details of soil chemistry composition in Supplementary Table 3.

### Experimental setup

Samples were incubated under 150 mg kg^−1^ of ammonium sulfate ((NH_4_)_2_SO_4_). The concentration of inorganic nitrogen was selected as it reflects the levels of nitrogen addition commonly used in the WA agricultural sector for soils as demonstrated in a prior study (Moreira‐Grez *et al*., 2019a). Briefly, 5 g of topsoil and 625 μl of sterilized water were added to a 120 ml serum bottle, aseptically. No other nutrient sources were added to avoid the enrichment of possible copiotroph communities that might hinder the growth of the targeted oligotrophic communities present in the study. Bottles were sealed with a rubber stopper and clamped to prevent air exchange throughout the experiment. Twelve replicates were prepared with destructive sampling over a 3‐weeks period (three replicates per timepoint). Bottles were incubated at 25°C in dark conditions. Soil from the topsoil storage facility was sampled and kept stored at −20°C until downstream analyses in order to assess baseline community composition (herein, timepoint zero).

### DNA extraction and 16S rRNA gene amplicon PCR

DNA was extracted in triplicate from 0.3 g of soil samples using a Powersoil‐htp 96 Soil DNA isolation kit, following the manufacturer's guidelines with minor modifications [freeze–thaw cycle (×3) after the addition of solution C1; 50 μl of solution C6 passed twice through the silica filter]. Extracted DNA was quantified using a Qubit 2.0 fluorometer (Life Technologies, USA). For the 16S rRNA analysis, 2 ng of DNA was used as template for subsequent PCR amplification using the 515F/806R primer set, targeting the 16S rRNA V4 region for both bacteria and archaea domains (Liu *et al*., 2007). For the in‐depth protocols, refer to Kumaresan *et al*. (2017) for PCR reagent and thermal conditions.

### 16S rRNA gene amplicon analysis

To identify relevant time points for shotgun metagenomic analysis, amplicon sequencing was used as a first step. PCR amplicons were sequenced using the Ion Torrent PGM platform (Thermo Fisher Scientific, Australia). Analysis was performed within QIIME wrapper (v. 1.9; Caporaso *et al*., 2010) using parameters described in the paper by Moreira‐Grez *et al*. (2019b). Briefly, low‐quality sequences were rejected (phred score <20, size between 130 and 350). No primer mismatch and/or barcode error were allowed. Chimeric sequences were filtered using USEARCH (v. 6.1; Edgar, 2010). Quality‐passed reads were then clustered using UCLUST4 at 97% sequence similarity cut‐off for the identification of representative OTUs. Taxonomic assignment of representative sequences was done using RDP classifier based on the Greengenes database (v. 13.8; McDonald *et al*., 2012). Resulting taxonomic frequency table was used for determining if notable community differences were observed between time points for further shotgun metagenome sequencing.

After considering the microbial community composition based on the 16S rRNA gene analysis, DNA extracted from the baseline community (time zero), 2‐ and 3‐weeks post‐enrichment replicates were selected for whole metagenome shotgun (WMS) sequencing, due to minimal differences in the relative abundance of the microbial communities between the baseline and 1‐week enrichment samples. Extracted DNA samples for WMS were sent to the Australian Genome Research Facility (https://www.agrf.org.au/) for sequencing. Library preparation was performed using the Takara ThruPLEX protocol (version: QAM‐108‐003) and sequenced via the HiSeq 4000 (Illumina) sequencing platform utilizing the 150 bp paired‐end technology.

### Functional and taxonomic profiling of soil metagenomes

Raw reads sequenced from the soil metagenomes were processed with Trimmomatic (v. 0.39; Bolger *et al*., 2014) to remove low‐quality bases and Illumina adapter sequences with the parameters ‘*PE –phred33 ILLUMINACLIP*:*TruSeq3‐PE*.*fa*:*2*:*30*:*10 LEADING*:*3 TRAILING*:*3 SLIDINGWINDOW*:*4*:*20 MINLEN*:*36*’. Reads in which both pairs passed the trimming process were kept for further analysis in the workflow. An estimation of coverage and diversity capture between the temporal samples was performed with Nonpareil (v. 3.3.4; Rodriguez‐R *et al*., 2018), using the alignment algorithm option via the *‘‐T alignment’* flag. Nonpareil was used to estimate required sequencing efforts needed to cover the calculated diversity between the baseline soil and post‐enrichment samples. The associated Nonpareil R package was used to plot the redundancy curves and report the estimated diversity index between the temporal samples.

Taxonomic profiling of microbial abundance was done at the read level using Kaiju (v. 1.7.4; Menzel *et al*., 2016), with default parameters using the ‘*nr_euk’* database (https://kaiju.binf.ku.dk/database/kaiju_db_nr_euk_2021-02-24.tgz) obtained from the Kaiju website. Outputs were combined from samples with the same time point (baseline, 2 and 3 weeks) for determining the taxonomic abundance at the phylum level via the *‘kaiju2table’* module using the *‘‐r phylum’* flag (Supplementary Table 4). Entries under *‘cannot be assigned to a (non‐viral) phylum’* and *‘unclassified’* were combined under the label *‘Unassigned/unclassified’*. Relative abundance computed at the phylum level with 16S rRNA amplicon sequences (QIIME) and metagenomic reads (Kaiju) were used to assess ammonium‐dependent enrichment over the 3‐week period. The eight most abundant phyla were selected for plotting on both datasets, although ‘*Unassigned/unclassified’* bin was also included for the metagenomic reads due to its large proportion throughout the dataset. Remaining taxa (other bacteria and archaea, which within the metagenomic subset also include eukaryotes and viruses) were classified as ‘other’. Stacked bar charts were computed using triplicates means and were visualized through the ggplot2 R package (v. 3.3.5; Wickham and Sievert, 2016).

For a more in‐depth analysis, the *‘kaiju2table’* module was also used to explore the effect of the enrichment on the differential abundance for all replicates across all samples (three each for baseline, 2‐ and 3‐weeks post‐enrichment) at the genus level using the *‘‐r genus’* flag (Supplementary Table 5). The genus‐level output from Kaiju was imported into R v. 4.1.0 and features were rarefied at 39292701 reads per sample, to allow for meaningful comparison when needed. Alpha‐ and beta‐diversity, between the soil community taxa spanning different time points was performed via the Shannon diversity index and NMDS analysis based on the Bray–Curtis dissimilarity coefficient respectively; implemented in the Vegan R package (v. 2.5‐7; Oksanen *et al*., 2020). Statistical difference between Alpha diversity values was tested using one‐way ANOVA with Tukey as a *post hoc* test as implemented on the multcomp R package (v. 1.4‐17; Hothorn *et al*., 2008). Resulting figures for the alpha‐ and beta‐diversity plots of microbial community structures were visualized by ggplot2.

Prokaryotes known to oxidize ammonia – AOA *Nitrososphaera*, *Nitrosocosmicus*, *Nitrosocaldus*, *Nitrosotalea*, *Nitrosopumilus Nitrosoarchaeum*, *Nitrosotenuis* and *Nitrosopelagicu*s; AOB *Nitrosomonas*, *Nitrosospira* and *Nitrosococcus*; and including comammox *Nitrospira*, were plotted to determine the effects of (NH_4_)_2_SO_4_ enrichment on the relative abundance of this subset of taxa. Corrplot R package (v. 0.90; Wei and Simko, 2021) was used to create a correlation matrix to analyse the strength of association between the relative abundance of AOA and AOB within the baseline soil and soil 3‐week post‐enrichment. The subsequent scatter and correlation plots for representing relative abundance and the correlation matrix respectively, between ammonia‐oxidizing prokaryotes were created using ggplot2.

Functional profiling of the metagenome was performed at the read level using SUPER‐FOCUS (v. 0.34; Silva *et al*., 2016), which groups metabolic genes into functionally similar groups based on a reduced SEED subsystem (Overbeek *et al*., 2005). SUPER‐FOCUS was run using default parameters, with DIAMOND (v. 0.9.14; Buchfink *et al*., 2015) as the aligner of choice among all individual samples. The Shannon diversity index was used to determine significant differences between metabolic genes at the Level 3 SEED subcategory comparing data from replicates from the baseline and 3‐week post‐enrichment samples through the Vegan R package with the results visualized by ggplot2. ANOVA‐like differential expression analysis was used to compare metabolic gene counts between the baseline and 3‐week post‐enrichment samples via the ALDEx2 v. 1.24.0 R package using the *‘t’* option for Welch's *t* and Wilcoxon rank test (Fernandes *et al*., 2013; Fernandes *et al*., 2014; Gloor *et al*., 2016). The conversion of metabolic count data through centred log scale transformation in ALDEx2 into a scale invariant form removed the need for prior rarefication of read counts and the transformed data underwent 128 Dirichlet Monte‐Carlo Instances and 10 000 iterations with Benjamini–Hochberg correction. Results from ALDEx2 were then visualized via a volcano plot using the EnhancedVolcano package (v. 1.12.0; Blighe *et al*., 2021). Genes with Log2 fold change lower than −2.5 and higher than 2.5 while having a significance level below alpha level (*α* < 0.05) were identified as significant microbial enrichments/suppressions (Supplementary Table 1).

### Genome recovery from soil metagenomes

*De novo* assembly of the metagenomes were performed using metaSPAdes (v. 3.15.1; Nurk *et al*., 2017) using default parameters and the *‘‐‐only‐assembler’* option enabled. MetaSPAdes was selected as the assembler of choice as it has been recommended for studies that aim to reconstruct representative genomes from the environment, which was one of the main focuses of this article (Vollmers *et al*., 2017). Experimental replicates within each temporal sample (baseline, 2 weeks and 3 weeks enrichment) were respectively co‐assembled to improve the rate of genomic recovery. Read mapping of the assembled contigs and MAGs were performed using Bowtie 2 (v. 2.3.4.1; Langmead and Salzberg, 2012). Postprocessing of read alignment files such as sorting and indexing of read maps for downstream analyses was carried out using Samtools (v. 1.10; Li *et al*., 2009). Binning was done using metaBAT2 (v. 2.12.1; Kang *et al*., 2019) using default parameters, with the quality and assembly statistics of the binned MAGs reported by CheckM (v. 1.1.2; Parks *et al*., 2015). Archaeal MAGs with reported completion >50% and <5% contamination were selected for further downstream analysis.

### Characterization of metagenome‐assembled genomes

MAGs were taxonomically classified via GTDB‐tk (v. 1.5.0; Chaumeil *et al*., 2020) using the Genome Taxonomy Database release 06‐RS202 data (Parks *et al*., 2020). Functional annotation of the MAGs was done using MetaErg (v. 1.2.3; Dong and Strous, 2019), with the associated database (released on Jan. 8, 2021, from: http://ebg.ucalgary.ca/metaerg/db.tar.gz). MetaERG is an annotation pipeline that utilizes various HMM and sequence search tools, as well as metabolic pathway prediction tools to automate the functional profiling of metagenomes and MAGs. Within MetaErg, output from Aragorn (v.1.2.41.c; Laslett and Canback, 2004) was used to predict tRNA and rRNAfinder (v. 1.1.0; Dong and Strous, 2019) was used for the 5S, 16S and 23S rRNA sequences for the purposes of MIMAG reporting (Bowers *et al*., 2017). MetaErg functional profile output results from HMMER (v. 3.1; Eddy, 2011) utilizing the Pfam‐A (Finn *et al*., 2014) and TIGRFAM (Haft *et al*., 2013) databases as well as the metabolic pathway prediction by MinPath (v. 1.5; Ye and Doak, 2009) for KEGG pathways (Kanehisa and Goto, 2000) were investigated to detect proteins related to nitrogen metabolism.

### Phylogenomic comparisons of MAGs and nitrogen pathways

To prepare data for genome‐wide phylogenetic comparison, proteins were predicted for each individual MAG via Prodigal (v. 2.6.3; Hyatt *et al*., 2010). Only archaeal MAGs classified by GTDB from canonical AOA groups were selected for comparison in phylogenetic and pathway distributions. These were then processed via PhyloPhlAn (v. 3.0; Asnicar *et al*., 2020), using the ‘*supermatrix_aa*.*cfg’* configuration file generated by ‘*phylophlan_write_default_configs*.*sh’*, which uses DIAMOND to map proteins to a marker database and MAFFT (v. 7.487; Katoh and Standley, 2013) for multiple sequence alignment. The database used was the PhyloPhlAn database via the ‘*‐d phylophlan*’ option, containing 400 universal markers that are detailed in the paper by Segata *et al*. (2013) with the *‘‐‐accurate’* option enabled. PhyloPhlAn was first run using default parameters to determine the minimum number of markers present in the genomes used in the study via the standard output using the *‘‐‐verbose’* option. As one MAGs returned only 99 markers, the minimum number of marker option was set using the flag ‘*‐‐min_num_markers 99*’. Archaeal MAGs recovered in the study were compared to reference genomes and as the highest level of taxonomy used to distinguish genomes was at the family level, the diversity setting was set to *‘‐‐diversity medium’*. Genomes used as comparative references for AOA candidate MAGs were from canonical AOA members and obtained from NCBI's Refseq database (Supplementary Table 6).

To create a bootstrapped tree, the concatenated alignment outputs for bacterial and archaeal marker genes produced by the Phylophlan pipeline were run separately through RAxML‐HPC (v. 8.2.12; Stamatakis, 2014) with the parameters *‘‐p 1989 ‐x 12345 ‐N 100 ‐m PROTCATLG ‐f a’*. The bootstrapped trees produced by RAxML‐HPC were visualized via iTOL (v. 6.3; Letunic and Bork, 2021). Visualization also included the presence of the nodes within selected KEGG pathways modules for nitrogen metabolism: Denitrification (M00529) and Complete nitrification (M00804) (Supplementary Table 7). In cases where the AmoCAB node was absent from Minpath predicted KEGG pathway, the functional profile created by MetaErg utilizing HMMER and the Pfam A database was used to confirm the presence of ammonia monooxygenase proteins.

## Author Contributions

D.K. and B.M.‐G. designed and performed the experiments. P.V. and B.M.‐G. performed the analyses. P.V. wrote the manuscript with contributions from D.K., B.M.‐G., M.J.W., A.S.W. and P.K. All authors read the manuscript and approved the content.

## Availability of Data and Materials

All sequencing data and genomes have been deposited at the National Center for Biotechnology Information (NCBI) under the accession number PRJNA775848.

## Supporting information


**Supplementary Table 1.** ALDEx2 output of significantly a) suppressed and b) enriched metabolic genes under the SEED subsystem level 3 subcategory from the baseline and three‐week post NH4 enrichment samples.
**Supplementary Table 2.** GTDB‐tk taxonomic classification, CheckM assembly statistics, predicted tRNA and rRNA of recovered archaeal MAGs.
**Supplementary Table 3.** Chemical characteristics of stored topsoil.
**Supplementary Table 4.** Taxonomic distribution of the metagenome at the read level from Kaiju. Relative abundance of phyla/taxa across combined treatment samples at each time point.
**Supplementary Table 5.** Taxonomic distribution of the metagenome at the read level from Kaiju. Read counts of taxa at the genus level across individual treatment samples at each time point.
**Supplementary Table 6.** RefSeq ammonia‐oxidizing archaea genomes used in study (Accessed 27/08/2021).
**Supplementary Table 7.** Nitrification and denitrification pathways predicted from a) recovered archaeal MAGs and b) RefSeq genomes, including CheckM completeness/contamination statistics.
